# Novel Ingredients: Hydroxytyrosol as a Neuroprotective Agent; What Is New on the Horizon?

**DOI:** 10.3390/foods14213624

**Published:** 2025-10-24

**Authors:** Lorena Martínez-Zamora

**Affiliations:** Applied Biology Department, Miguel Hernández University, Avda. de la Universidad, s/n, Ed. Vinalopó, 03202 Elche, Spain; lorena.martinezz@umh.es

**Keywords:** mediterranean Diet, cognitive function, olive oil, polyphenols, brain, neuroinflammation, oxidative stress, antioxidant activity, functionality

## Abstract

Hydroxytyrosol (HXT), a phenolic compound from olive, shows great potential as a neuroprotective agent and a translational target for claim-ready nutrition and food products. Human studies increasingly report benefits for vascular function, inflammatory tone, and early cognitive/psychomotor outcomes, consistent with engagement of redox and signalling pathways (Keap1–Nrf2–ARE, PI3K/Akt–ERK, and AMPK–SIRT1–PGC-1α). HXT is rapidly absorbed and likely reaches the brain, acting on endothelial and microglial targets. On the neurovascular axis, it reduces oxidative stress, preserves nitric-oxide bioavailability, lower inflammatory markers, and favourable intrinsic connectivity. For product development, bitterness from oleuropein-rich inputs can be mitigated by hydrolysis, followed by structure-guided delivery to balance sensory quality with exposure. Viable formats include cyclodextrin inclusion, microencapsulation, and (micro)emulsions in lipid matrices, plus stability engineering for aqueous systems (acidification, chelation, low-oxygen handling, or barrier packaging). Matrix effects are consequential; some proteins and fibers may decrease HXT bioaccessibility, whereas lipid phases and microstructured carriers often enhance it. Clinically, recommended doses are ~7–15 mg/day chronically and ~30–60 mg acutely. As conclusions of this review, future work should prioritize harmonized pharmacokinetics–pharmacodynamics readouts, cognition anchored to a compact neurovascular/blood–brain barrier biomarker core, and head-to-head comparisons of manufacturable delivery formats.

## 1. Introduction

Food is an essential part of our lives, transcending its basic role of providing nutrients necessary for human survival. It significantly shapes cultural identities, and it profoundly impacts human health. Furthermore, the connection between dietary habits and mental health is widely known [1,2,3]. As a matter of fact, neurotransmitters like dopamine and serotonin, which play a crucial role in regulating mood and emotions, can be influenced by what we eat. A diet rich in essential nutrients, including omega-3 fatty acids, B vitamins, and amino acids, supports the production of these neurotransmitters [4]. Following a nutritious, well-balanced diet has been found to lower the risk of depression [5], whereas consuming diets high in processed and sugary foods and low in fiber and healthy fats correlates with a higher risk of depression [6,7,8,9].

The Mediterranean diet (MD), known for its health benefits, emphasizes the consumption of fruits, vegetables, whole grains, vegetable proteins, and healthy fats like extra virgin olive oil (EVOO) [10,11], whose consumption has been directly related to the reduction of mental illness [4]. As one of the main ingredients of MD, around 3 M tons of olive oil are annually produced worldwide. Knowing that approximately 4–6 kg of olives are needed to produce 1 L of olive oil, its production generates between 3–5 kg of olive by-products [12], which at a global level would mean a waste of 12 M tons per year.

One of the most important phytochemicals found in olive by-products is hydroxytyrosol (HXT), which has garnered attention during the last twenty years due to its important health benefits. HXT is a powerful antioxidant whose consumption offers several health effects, including cardiovascular, anti-inflammatory, neuroprotection and cognitive health, anti-cancer, skin protection, immune system support, anti-microbial, and anti-diabetic [13,14]. HXT is associated with neuroprotective effects, which have prompted research into its potential to prevent cognitive decline, particularly in relation to Alzheimer’s disease [15]. This neurodegenerative disorder is the most common form of cognitive impairment associated with aging in Western societies, characterized mainly by a gradual decline in memory that affects daily activities.

**Figure 1:**
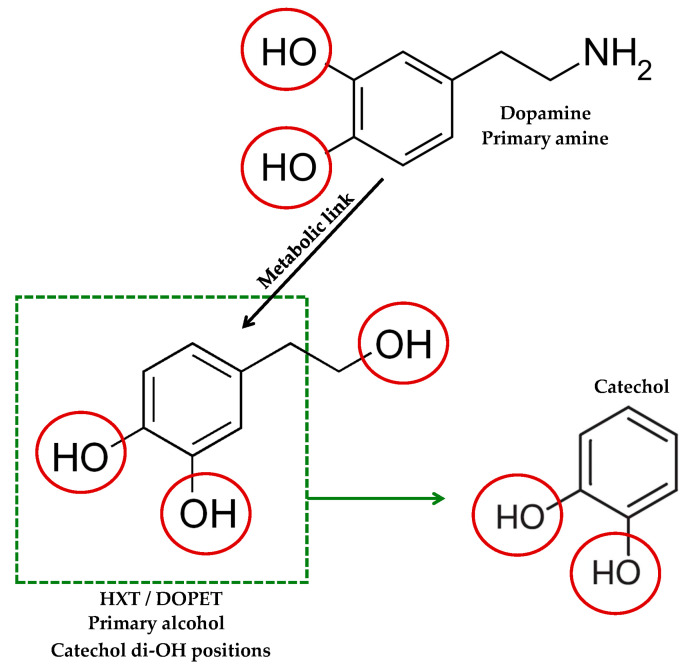
HXT vs. dopamine and catechol. Structural comparison highlighting the catechol di-OH positions and side-chain functionalities.

Consistent with these properties, HXT (3,4-dihydroxyphenethyl alcohol) is a small phenylethanoid featuring a catechol ring and a primary alcohol side chain. Its high polarity and multiple hydrogen-bond donors/acceptors confer good aqueous compatibility alongside moderate lipophilicity, so it remains water-compatible while still engaging lipid environments relevant to cell membranes [13]. In physiological media, the phenolic groups remain largely unionized. The catechol can be reversibly oxidized to an o-quinone, which supports radical scavenging and metal chelation. Together, this polarity and catechol redox behaviour explain the reactivity of HXT with ROS and its indirect antioxidant actions (Figure 1).

Clinical and experimental evidence further supports the role of olive oil phenolic compounds, including HXT, in combating age-associated cognitive decline. Recently, Boronat et al. [16] reviewed several studies indicating that the antioxidant properties of these compounds help mitigate oxidative stress and inflammation, two key factors in the progression of neurodegenerative diseases like Alzheimer’s in humans. However, the bioavailability of HXT when it is incorporated as ingredient in foods and its potential for neuroprotective ability has not been reviewed before. The consumption of olive oil rich in HXT has been associated with improved cognitive performance in older adults [16,17], highlighting its neuroprotective effects. This evidence suggests that regular dietary intake of HXT can have a substantial impact on maintaining cognitive health also in aging populations.

Numerous requests have been made to the European Food Safety Authority (EFSA) Panel on Dietetic Products, Nutrition, and Allergies to evaluate HXT, including its chemically synthesized form, as a novel ingredient under Regulation (EC) No 258/97 [18]. Similarly, the Food and Drug Administration (FDA) has been approached regarding its use as an antioxidant food additive and in the preparation of functional foods [19]. Both EFSA and the Spanish Agency for Consumer Affairs, Food Safety, and Nutrition (AESAN) have deemed it safe for proposed uses and levels. The FDA considers HXT safe in processed foods at a level of 5 mg per serving, with a maximum exposure of 51.06 mg per person daily [20].

Therefore, the main objective of this review is to articulate an integrated, translational framework that links mechanistic evidence to clinically actionable claims and manufacturable food formats for HXT in mental-health nutrition. To this end, five specific objectives will be pursued: (i) to summarize HXT pharmacokinetics; (ii) to synthesize evidence across neurovascular, neuroimmune, and dopaminergic pathways; (iii) to examine clinical trials using HXT-rich foods or standardized preparations; (iv) to address the main strategies used to produce HXT-rich foods; and (v) to set out research and future perspectives to advance HXT from plausible mechanisms to real functionality as neuroprotector agent.

## 2. Pharmacokinetics and Brain Bioavailability of Hydroxytyrosol

**Figure 2:**
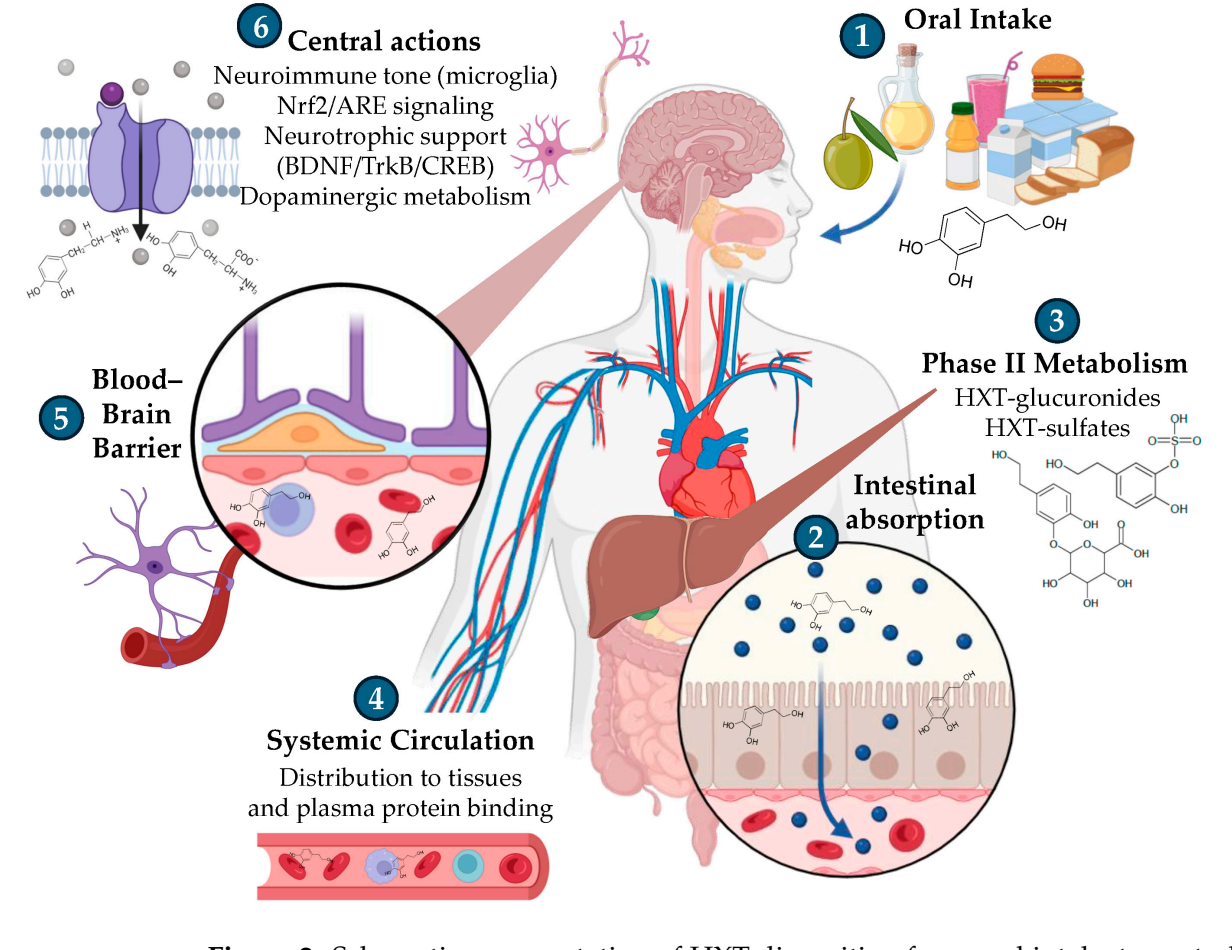
Schematic representation of HXT disposition from oral intake to central targets. Figure created with assistance from BioRender (https://biorender.com/; accessed on 5 September 2025), image concept, improvements, and curation performed by the author.

After ingestion, HXT is absorbed in the intestine, undergoes extensive Phase II metabolism in the liver (glucuronidation and sulfation), and circulates systemically mainly as these conjugated forms. Through systemic circulation, there is evidence that HXT and its metabolites can cross the blood–brain barrier (BBB) (Figure 2), although the specific transporters and permeability routes remain to be fully elucidated. Within brain regions such as the hippocampus and cortex, central actions include modulation of neuroimmune tone (microglial activity), activation of the Keap1/Nrf2/ARE pathway, support of neurotrophic signalling that regulates the memory-cognition-mood axis (BDNF, TrkB receptor, and CREB), and interactions with dopaminergic metabolism [21,22].

In a specific way, starting from oral intake, either as a natural constituent of olive oil or as purified HXT used as ingredient, it is rapidly absorbed in the small intestine and undergoes extensive Phase II metabolism in enterocytes and the liver [23,24] (Figure 2). Human and animal studies consistently show that glucuronide and sulfate conjugates dominate the circulating pool, while free HXT is transient and typically low or undetectable in plasma with standard handling, underscoring the need to quantify conjugates and catechol-O-methylated forms (e.g., homovanillyl alcohol, homovanillic acid, etc.) [23].

In this sense, recent studies have shown that matrix effects are relevant in humans, since bioavailability differs when HXT is consumed within olive oil vs. refined oils or dairy matrices [21]. On the other hand, supplementation studies confirm dose-dependent absorption with predominance of HXT-3-O-sulfate and glucuronides in plasma and urine [23,25]. In addition, red blood cells may act as transient carriers that modulate distribution of phenolic conjugates [24,26].

Classical and modern pharmacokinetics studied by LC-MS/MS indicate rapid systemic appearance (maximum absorption within the first hours after consumption) and largely linear kinetics over practical dose ranges [21,23]. In rodents, recent studies using HXT delivered in table olives showed linear pharmacokinetic between ~3–6 mg/kg with robust detection of sulfate and glucuronide conjugates. These data complement earlier human/animal excretion studies that highlighted species differences and high urinary recovery of HXT metabolites within 24 h [21,22].

Once HXT metabolites have reached the systemic circulation, direct brain access has been explored with in vitro BBB models and in vivo studies. In co-culture systems based on human brain endothelial cells (hCMEC/D3) [27], HXT showed trans-endothelial transport compatible with BBB passage and exerted protective effects under oxidative stress, which supports feasibility of central exposure [28]. Complementary BBB models from 2024–2025 refine transporter expression and barrier tightness, providing platforms to test HXT and its conjugates systematically [29], although it has not been studied with HXT yet.

The amphiphilic architecture of HXT (catechol headgroup + short alkyl chain) lets it briefly interact with phospholipid membranes and, when gradients allow, passively cross their hydrophobic core. Membrane organization modulates the process to more fluid membranes facilitating lateral movement, whereas lipid rafts can transiently concentrate catechols via π–π stacking and hydrogen bonds, modulating uptake in specific membrane microdomains. In vitro BBB and cell-based models (MDCK, hCMEC/D3) consistently indicate low-to-moderate passive permeability, compatible with measurable but limited brain entry [27]. Phase-II conjugation (sulfation/glucuronidation) increases polarity and can reduce passive BBB flux. These conjugates may still reach the central nervous system through transport-mediated routes and/or local deconjugation. Finally, efflux transporters (e.g., P-gp/MRP) could limit net central nervous system accumulation.

In vivo, regional brain pharmacokinetic data in depressive-like mice demonstrated that HXT crosses the BBB and accumulates in the brain, with hippocampus region showing the highest exposure [30]. However, BBB permeability changes in the disease model, which appeared to facilitate entry [30]. The same work linked regional HXT exposure to microglial C3-CD11b pathway modulation, suggesting mechanistic relevance., while most measurements refer to total HXT (often after deconjugation). These studies substantiate BBB penetration in rodents [30], although these kinds of studies have not been carried out in humans, due to the difficulty of sampling and the ethical issues involved.

Emerging investigations are beginning to ask whether conjugated metabolites (sulfates and glucuronides) themselves can cross the BBB and/or be deconjugated locally [31,32]. Recent pharmacology papers on phenolic metabolites and BBB transport outline tools to quantify brain-to-plasma ratios and perfusion effects, but definitive transporter identities for HXT conjugates in humans remain to be clarified [31,32].

Therefore, the best current evidence for regional brain distribution comes from rodent work demonstrating preferential hippocampal localization after oral HXT consumption, with region-specific molecular responses [30]. Moreover, previous authors have shown that some models are not directly comparable between rats and humans, for example, urinary excretion [33], which is closely linked to bioavailability and therefore to potential brain penetration. These data converge with broader reviews noting detection of HXT (or its derived phenolics) in brain tissue and the plausibility of interactions with catecholaminergic pathways, though high-resolution human mapping is not yet available [14,34,35].

## 3. Neurovascular and Neuroimmune Pathways

HXT interfaces with cerebrovascular biology at three clinical levels: (i) it lowers vascular oxidative stress and supports endothelial function, (ii) it helps preserve BBB homeostasis, and (iii) it improves BBB permeability, functional connectivity, and cognition, which has been corroborated in human studies with phenolic-rich olive foods.

**Figure 3:**
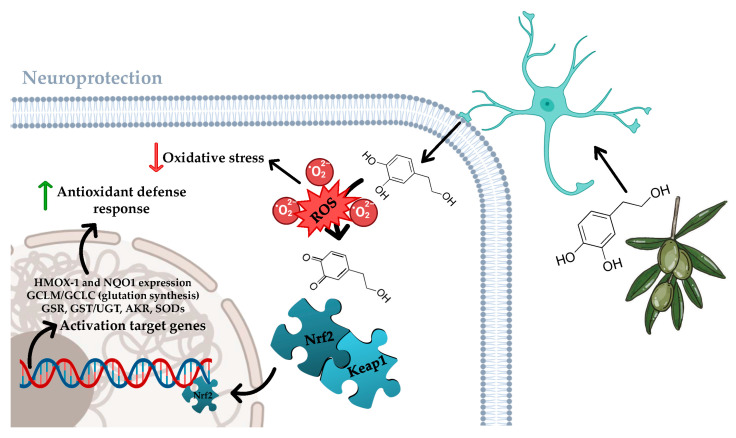
Neuroprotective pathways regulated by HXT in brain and mood function. Figure created with assistance from BioRender (https://biorender.com/; accessed on 10 September 2025), image concept, improvements, and curation performed by the author.

Across vascular beds, HXT is a potent scavenger of reactive oxygen species (ROS) and modulator of redox-sensitive signalling that converge on endothelial nitric oxide (NO) bioavailability [36,37]. In human endothelial in vitro models, HXT has demonstrated that it can reduce mitochondrial and NADPH oxidase, derived ROS, preserve NO, and limit leukocyte adhesion through down-regulation of adhesion molecules (ICAM-1/VCAM-1) under inflammatory stimuli [36]. Mechanistically, activation of PI3K/Akt/ERK and SIRT1–AMPK axes lead to Nrf2 nuclear translocation throughout the Keap1/Nrf2/ARE pathway with induction of HMOX-, NQO1, and SODs, supporting cytoprotection and wound repair [38,39] (Figure 3). However, direct endothelial NO synthase phosphorylation responses may vary by model and dose [37]. Post-prandially, high-polyphenolic EVOO acutely improves brachial flow-mediated dilation vs. refined olive oil in adults at cardiometabolic risk, consistent with a polyphenol-dependent endothelial signal [40]. Taken together, these data position HXT as a lead phenolic for maintaining endothelial homeostasis against oxidative and inflammatory stressors relevant to neurovascular damage.

Tight-junction architecture and adherent functions are key BBB determinants that are degraded by cytokine- and ROS-driven signalling [41]. In vitro BBB platforms (e.g., hCMEC/D3) show that HXT reduces oxidative-stress injury to brain endothelial cells and, in co-culture with SH-SY5Y neurons, lessens Parkinson-related stress, which supports feasibility for direct protection of the BBB [42].

Classical intercalation is unlikely for HXT because its molecular structure is small and non-planar compared with polyaromatic intercalators. Even so, the catechol group (Figure 1) supports two complementary genoprotective actions: scavenging of reactive species (•OH) that generate 8-oxo-dG and related lesions, and bidentate chelation of Fe/Cu. Consistently, studies in cells and animals report lower markers of oxidative DNA damage with HXT or olive phenolics [38,39]. Although evidence for DNA adduct formation or non-intercalative surface binding is limited, targeted spectroscopic assays (UV–Vis, fluorescence displacement, circular dichroism) together with molecular dynamics/docking could clarify potential weak and non-intercalative binding modes.

Clinically, a randomized trial in mild cognitive impairment found that 6-month EVOO consumption vs. refined olive oil (ROO) decreased hippocampal/parahippocampal gadolinium extravasation quantified by DCE-MRI (primary outcome), indicating improved BBB function [17]. In this study, Kaddoumi et al. also showed that EVOO enhanced resting-state functional connectivity [17]. These findings align with the literature where subtle BBB leakage is quantifiable by dynamic contrast and magnetic resonance imaging, and may be tracked with complementary approaches (e.g., BBB-ASL) [43,44].

In the MICOIL pilot randomized clinical trial [45], the consumption of high-phenolic EVOO (50 mL/day) improved multiple cognitive domains (ADAS-Cog, MMSE, working memory/fluency) in comparison with moderate-phenolic EVOO and MD advice alone across 12 months [45]. Also, the Spanish randomized clinical trial of 6.5 years, PREDIMED-Navarra, reported higher MMSE and Clock-Drawing scores in participants assigned to MD+EVOO compared to the low-fat control diet, suggesting long-term cognitive benefits [1]. The Kaddoumi et al. randomized clinical trial [17] found EVOO reduced BBB permeability and increased functional connectivity compared to ROO in mild cognitive impairment (6 months). Additionally, EVOO consumption also reduced neurofilament light, indicating a neuroaxonal protection signal. Collectively, these trials support a plausible chain from phenolic exposure to endothelial and BBB effects, as well as cognitive/mood endpoints.

In the case of neuroimmune pathways, microglia are key transducers of diet-derived signals into neuroimmune tone. In vitro studies in BV2 and primary microglia cell lines showed as HXT suppresses LPS- and α-synuclein-induced activation by inhibiting NF-κB p65 nuclear translocation, reducing NOX2 activity, and lowering TNF-α, IL-1β, IL-6, iNOS, and COX-2 [46]. In α-syn contexts relevant to Parkinson’s disease, HXT reduces the TLR4/MyD88 cascade that drives NF-κB/MAPK signalling and NLRP3 inflammasome activation, a central pathogenic axis produced during Parkinson’s disease [42].

Also related to the neuroprotector function of HXT, this compound robustly engages the Keap1-Nrf2-ARE pathway across endothelial and neural tissues. Early mechanistic work demonstrated HXT-evoked Nrf2 translocation via PI3K/Akt and ERK, with induction of HMOX1/NQO1/SOD and cytoprotection against oxidative stress [38,39], as previously shown in Figure 3. In vivo, HXT activates AMPK–SIRT1–PGC-1α and up-regulates Nrf2 targets in brain, improving mitochondrial complex I/IV expression and lowering oxidative protein damage [47]. In this sense, comparative studies indicate HXT and acylated derivatives converge on Nrf2 activation as a shared cellular pathway [48]. Given the established role of astrocytic Nrf2 in neuroprotection [49], this pathway is a plausible mediator of HXT’s central effects.

On the other hand, across innate-immune cascades that drive neuroinflammation, HXT consistently dampens pro-inflammatory cytokine networks initiated by pattern-recognition receptors. In BV2 microglia and mixed neurovascular in vitro cell cultures, HXT suppresses TLR4–MyD88 signalling, down-modulating NF-κB p65 nuclear translocation and ERK/MAPK activation, with downstream reductions in TNF-α, IL-1β, IL-6, iNOS, and COX-2 expression [46]. These effects accompany a phenotypic shift from M1-like to M2-like microglia and tighter cytokine control under oxidative stress [46].

Beyond proximal TLR signalling, HXT and derivatives blunt inflammasome activation. Although much of the mechanistic mapping comes from peripheral and ex-vivo models, convergent evidence points to attenuation of the NLRP3–caspase-1–GSDMD axis and IL-1β/IL-18 maturation, whose pathways now are recognized as central to neurodegeneration and mood-relevant neuroinflammation [50]. In microglial contexts where mitochondrial stress fuels inflammasome activation, HXT butyrate prevents sleep-deprivation-induced release of mitochondrial DNA from microglia, curbing ROS-dependent NF-κB/NLRP3 signalling and cytokine surges [51].

Furthermore, cytokine networks intersect with dopaminergic vulnerability and synaptic function. HXT reduces α-synuclein-triggered microglial activation and LPS-induced cytokine release, highlighting interference with dual infectious triggers of neuroinflammation relevant to Parkinson’s disease and mood-circuit pathology. Related tyrosols also limit α-syn aggregation and toxicity in neuronal and *Caenorhabditis elegans* models, which indirectly diminishes microglial cytokine amplification [42].

Finally, the JAK/STAT pathway integrates IL-6 family signalling, which is implicated in depression and cognitive decline. Although direct modulation of brain STATs by HXT has not been demonstrated, upstream suppression of IL-6/NF-κB signalling and attenuation of inflammasome-derived IL-1β and IL-18 would be expected to shift microglial STAT1/STAT3 programmes away from pro-inflammatory dominance toward pro-resolving states [52].

Therefore, knowing that neuroinflammatory cytokines (IL-1β, IL-6, TNF-α) perturb hippocampal plasticity and monoaminergic tone, and HXT’s cytokine control hence aligns with mood and cognitive end-points. In chronic stress paradigms, HXT (or HXT-rich food) reduces cortical/hippocampal cytokines, restores antioxidant defences, and improves depressive-like behaviour while reactivating BDNF/TrkB/CREB signalling, which is the main pathway for antidepressant responses [53].

When studying sleep-loss, a highly translational driver of mood symptoms, microglial DNA-mediated neuroinflammation is provoked, which is directly related to the dopaminergic function of this compound. In this case, HXT butyrate prophylaxis prevents this cascade, nominating an actionable route to protect mood-relevant circuits under real-world stressors [51].

At the clinical–behavioural interface, dietary patterns enriched in EVOO and polyphenols have reduced depressive symptoms in randomized trials (e.g., SMILES [54] or AMMEND [5] in young men) reporting benefits of EVOO itself in major depression, consistent with a role for HXT-rich matrices as co-adjuvants. While these trials are not HXT-monotherapy studies [5,54,55], they support translational plausibility that HXT-containing foods contribute to mood improvement through neuroimmune and trophic mechanisms.

Mechanistically, reducing cytokine noise protects long-term potentiation and preserves synapse-associated proteins; it also lessens dopaminergic stress in mesolimbic reward circuits, which are sensitive to IL-1β/IL-6 elevations [53,56]. Beyond inflammatory control, HXT’s tuning of redox status and mitochondrial function further shifts the microglial toward a neurotrophic profile, fostering resilience against mood deterioration and cognitive decline.

## 4. Dopaminergic Pathways and Motivation

Regarding its chemical structure, HXT is identical to 3,4-dihydroxyphenylethanol (DOPET), a minor endogenous metabolite of dopamine produced when the reactive catechol-aldehyde DOPAL is reduced by cytosolic aldehyde/aldose reductases (AR/ALR) [57]. This “reductive pathway” operates in parallel to the aldehyde dehydrogenase (ALDH) route that oxidizes DOPAL to DOPAC [57,58]. Although quantitatively minor under basal conditions, the DOPAL→DOPET reduction is considered a compensatory detoxification pathway within dopaminergic neurons and increases when ALDH activity is constrained by oxidative stress or xenobiotics [59,60]. In humans, endogenous DOPET formation is detectable and can rise with ethanol exposure [61], which elevates catecholamine turnover. Conversely, exogenous HXT from EVOO contributes to the same circulating analyte, complicating source attribution in biomarker studies [61].

Functionally, DOPET (and also HXT) displays dual relevance to dopamine homeostasis. On one side, antioxidant activity that limits spontaneous dopamine-quinone formation and catechol-aldehyde adducting and, on other side, feedback modulation of catecholamine synthesis via tyrosine hydroxylase inhibition at micromolar concentrations in cell systems, which is a potentially protective brake during oxidative load [62]. These properties position HXT as both a dopamine-pathway by-product and an active modulator of dopamine redox stress [62].

From a translational perspective, exogenous HXT consumed through diet or supplementation adds to the same analytical pool as endogenous DOPET. In this sense, two implications should follow for clinical work. On one hand, exposure assessment should quantify Phase-II conjugates of HXT and record alcohol intake (a confounder of DOPET) and, on the other hand, mechanistic readouts should incorporate indices of catecholaldehyde stress alongside standard dopaminergic biomarkers [61,63].

Across stress- and toxin-based models, HXT improves motivation-related behaviours while engaging molecular programs plausibly relevant to mesolimbic function. In chronic-stress paradigms, HXT increases sucrose preference and reduces immobility, is concomitant with lower IL-1β/IL-6/TNF-α, and it enhances the activation of Nrf2/HMOX1 and the up-regulation of BDNF/TrkB/CREB, which is a signature that aligns with improved synaptic plasticity within reward circuitry [38,39]. In dopaminergic cell models, HXT and derivatives attenuate 6-OHDA injury via Nrf2/HMOX1, supporting direct mitigation of dopaminergic oxidative stress [58,59,60]. Pharmacokinetics in rodent work further indicates hippocampal-biased brain exposure with microglial pathway modulation, providing an additional mechanistic bridge between immune tone and motivation [64]. While operant reward tasks remain underexplored for HXT, the convergence of behavioural antianhedonic effects, dopamine-relevant redox control, and neurotrophic signalling supports a working model in which HXT stabilizes mesolimbic plasticity under oxidative load [53].

## 5. Clinical Evidence and Translational Insights

Across randomized-controlled trials, increasing exposure to HXT (via purified HXT, HXT-enriched olive oils, or HXT-rich foods) consistently shifts vascular redox tone, endothelial phenotype, and low-grade inflammation in directions consistent with improved cerebral perfusion, BBB stability, and neuroimmune de-biasing. For instance, recent research has highlighted the significant impact of HXT on alleviating cognitive decline, particularly through its modulation of neurotrophic and inflammatory factors. Liu et al. [56] demonstrated that HXT (>25 mg/kg/day) alleviates obesity-induced cognitive decline in mice by modulating the expression levels of BDNF and inflammatory markers. BDNF is crucial for the survival, development, and function of neurons, and its increased expression is linked to enhanced cognitive function. By reducing inflammation and promoting neurotrophic support, HXT plays a protective role against cognitive impairment associated with obesity, underscoring its potential in mitigating neuroinflammatory conditions that contribute to cognitive decline. However, this kind of research must also show positive results in humans to demonstrate the functionality of this promising compound. In this sense, Table 1 summarizes the latest human findings on HXT’s neuroprotective effects and on cardiovascular or inflammatory effects that may directly influence neural function.

Across described human studies, improvements in vascular redox balance, endothelial function, and low-grade inflammation are observed with chronic intakes around ~7–15 mg HXT/day, while single-dose (acute) exposures of ~30–60 mg have been used to probe short-term vascular and oxidative responses. These ranges appear to be well tolerated and can be reached either via purified HXT or high-phenolic EVOO, depending on phenolic content and serving size. Importantly, matrix and formulation (e.g., lipid phase, microemulsions, cyclodextrin complexation) can meaningfully increase apparent exposure relative to aqueous vehicles. For product specification, the EFSA health-claim threshold (≥5 mg HXT/day with olive phenolics) provides a pragmatic lower limit, whereas higher targets within the ranges mentioned above may be preferable in cardiometabolic-risk subgroups, showing greater inflammatory burden [18]. Future trials should report both free and conjugated HXT metabolites, specify the phenolic profile of the intervention, and stratify outcomes by baseline inflammatory/oxidative status.

Directly related to the neurological protection ability of HXT, García-Layana et al. [77] showed that circulating HXT phenolics are captured and compliance-tracked (urinary and serum metabolites), while exploratory outcomes supported anti-inflammatory and vascular benefits that conceptually translate to brain microvessels being able to retard the macular neurodegeneration [77].

This vascular-immune re-balancing appears to matter for brain structure and function. In pregnant individuals enrolled in IMPACT-BCN, greater adherence to a MD pattern (which elevates urinary tyrosol and HXT) is related to thicker cortex in the praecuneus and left superior parietal lobule. Importantly, urinary tyrosol and HXT were positively associated with global mean cortical thickness, a structural proxy of brain integrity that generalizes to later cognitive resilience [81].

Beyond structure, a randomized, double-blind placebo-controlled trial of desert olive tree pearls rich in HXT and oleuropein reported improvements in composite memory and psychomotor speed on COGNITRAX after 8 and 12 weeks, which is consistent with enhanced attention–memory coupling and motor–cognitive integration in old adults (51–82 years old) [79].

Observational analyses from DIRECT-PLUS (a randomized dietary intervention with graded polyphenol arms) linked higher urinary HXT to a slower DNA-methylation pace of aging (DunedinPACE), suggesting systemic deceleration of biological aging processes tightly tied to neurodegeneration trajectories [80].

An open-label pediatric pilot in mitochondrial disease, especially with MELAS (mitochondrial encephalopathy, lactic acidosis, and stroke-like episode) found HXT supplementation was well tolerated over 12–18 months and associated with the largest gains in health-related quality of life (PedsQL). In addition, signals of benefit were observed in MELAS subgroups, consistent with HXT’s mitochondria-supporting Nrf2-linked mechanisms relevant to neuroimmune and synaptic homeostasis [82].

In mild Alzheimer’s disease, a 6-month randomized trial of an olive-leaf extract beverage (rich in oleuropein with downstream HXT generation) maintained global cognition and showed directionally favourable changes across functional scales and neuropsychiatric symptoms, with an unchanged depression score. Although not an HXT-only intervention, the phenolic-rich diet (MD) used here is metabolically connected to HXT exposure and mirrors the endothelial and inflammatory improvements seen in EVOO trials, making the neurocognitive signal biologically coherent [78].

The clinical signal extends to patient groups with high vascular-inflammatory burden. In older adults after myocardial infarction, randomized intake of high-phenolic EVOO (rich in HXT) improved overall neuropsychiatric function and lowered arterial glycocalyx injury (lower perfused boundary region) while enhancing brachial flow-mediated dilation [83].

**Figure 4:**
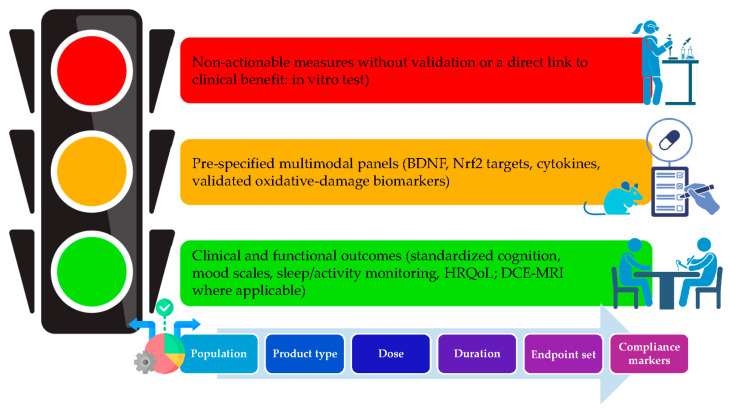
From mechanism to claims—endpoint hierarchy and trial-design decision tree. BDNF: Brain-Derived Neurotrophic Factor; Nrf2: Nuclear factor erythroid 2; HRQoL: Health-Related Quality of Life; DCE-MRI: Dynamic Contrast-Enhanced Magnetic Resonance Imaging. Figure created with assistance from BioRender (https://biorender.com/; accessed on 14 September 2025), image concept, improvements and curation performed by the author.

Taken together, human trials using HXT-centred strategies demonstrate a reproducible trial, including vascular improvement (FMD, PWV, CFR, PBR), inflammatory down-tuning, and early cognitive/psychiatric benefits (memory composites, psychomotor speed, MMSE maintenance), that aligns with the mechanistic picture of Nrf2-anchored redox control, endothelial barrier protection, and microglial de-biasing toward pro-resolution states. This provides a credible translational bridge from molecular pathways (Section 2, Section 3 and Section 4) to claim-ready endpoints (cognition-mood disorders and cerebrovascular function), while highlighting compliance markers (urinary HXT or tyrosol) and multi-omics panels (Nrf2-targets, oxidative-damage markers) to be embedded prospectively in future clinical trials, as summarized in Figure 4.

Mechanistically, the above human endpoints can be directly related to preclinical evidence asserting that HXT activates Nrf2/ARE and PI3K/Akt, collectively supporting synaptic proteins and LTP-like plasticity. The vascular randomized clinical trials showing improved FMD/CFR, reduced PBR, and lower oxLDL/CRP provide clinical-scale correlates of the following mechanistic nodes: more NO signalling, less endothelial activation (ICAM-1/VCAM-1 programs), and quieter cytokine noise. In parallel, HXT’s catechol structure links to dopamine biochemistry: as DOPET, it sits within catecholamine turnover and can lessen catechol-quinone/aldehyde stress, a contributor to mesolimbic and nigrostriatal dysfunction [83]. Human signals of improved psychomotor speed and mood are consistent with protection of dopaminergic circuits under inflammatory and oxidative pressure [83].

## 6. Strategies for Developing Neuroprotective HXT-Enriched Foods

**Figure 5:**
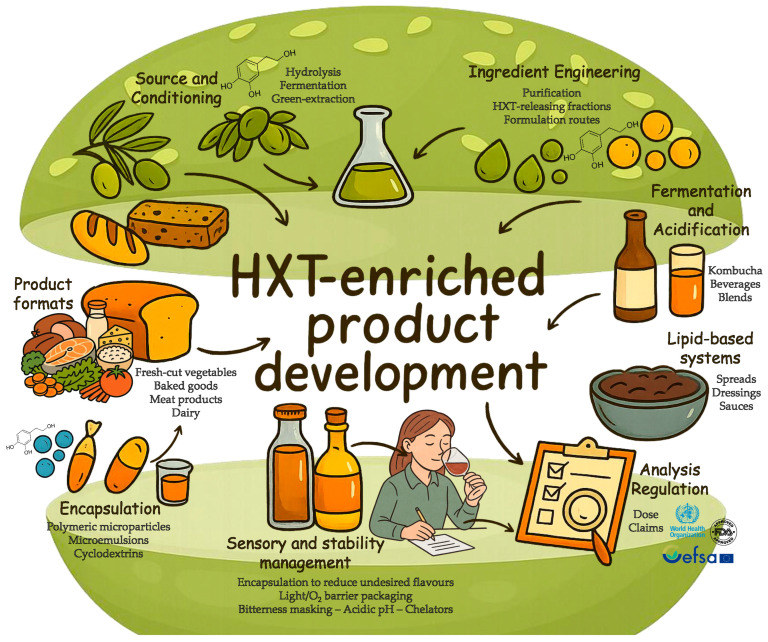
Strategies for developing and marketing HXT-enriched foods. Sora (https://sora.chatgpt.com/; accessed on 18 September 2025) has been used to assist in image generation by creating icons, image concept, improvements, and curation performed by the author.

Developing ready-to-eat foods enriched in HXT begins with ingredient sourcing (Figure 5). The most scalable inputs are olive-mill side streams, especially olive leaves, whose phenolic profile is dominated by oleuropein and HXT, imparting a distinctly bitter taste. Oleuropein and HXT impart a distinct bitter profile that can challenge acceptability in HXT-enriched foods. In this sense, sensory studies in beverages and bakery matrices show that bitterness is detectable and often dominant, yet acceptable when applying bitterness-mitigation strategies, such as cyclodextrin complexation, microencapsulation, and microemulsions, and when pairing with compatible flavor profiles (e.g., citrus, cocoa, robust cereal notes).

In line with the table-olive debittering process, where routine hydrolytic conversion of oleuropein into HXT and elenolic derivatives reduces bitterness, the same principle is applied to food-grade olive-leaf extracts intended for fortification [84]. This process consists of controlled hydrolysis before formulation both decreases perceived bitterness (without reducing phenolic functionality) and raises the free HXT fraction to most effective levels. Since the effectiveness of this step depends on the choice of catalyst, time, temperature, and downstream purification, its optimization largely determines the technological performance and consumer acceptance of HXT-enriched products [84]. Accordingly, incorporating a sensory validation step in product development (trained panel and consumer acceptability) is highly recommended.

In practice, the most selective route is β-glucosidase-assisted hydrolysis, which cleaves the glycosidic bond under mild conditions and has been advanced by targeted enzyme discovery and optimization; microbially driven bioprocessing and solid-state fermentations of olive leaves offer complementary, clean-label approaches that can simultaneously modulate taste and increase HXT or HXT-releasing derivatives in the resulting ingredient [84].

Once a suitably hydrolysed input is secured, formulation work naturally separates into stability management, sensory management, and delivery design. For instance, according to Kranz et al. [85], trained-panel sensory studies in beverage matrices report low detection and recognition thresholds for olive-leaf-derived bitterness, while acceptance improves when the matrix, and especially the sweet–acid balance, is deliberately tuned. Taken together, these results argue for early, matrix-specific threshold testing and the strategic use of bitterness-masking approaches when formulating with free HXT or minimally processed leaf extracts [85].

A powerful way to decouple in-mouth impact from delivered dose is encapsulation or molecular inclusion. Cyclodextrin inclusion complexes of HXT produce white, neutral-tasting powders with improved thermal robustness and controlled intestinal release, enabling higher formulation levels with minimal sensory penalty and providing a convenient, dose-standardized ingredient. Their technological feasibility is underscored by successful incorporation into wheat bread tested in humans without compromising acceptability [84,86]. Additionally, other authors have also demonstrated the effective incorporation in HXT-enriched food products without compromising the sensory acceptance, as in meat products [87,88,89,90,91,92], fish products [93], bakery products [86,94], salad dressing [95], or beverages [85], among others food [96].

Polymeric microencapsulation, for example with ethyl-cellulose microparticles produced by double-emulsion solvent evaporation, further protects HXT and delivers controlled release under simulated digestion, a format well-suited to dry mixes, instant beverages, and bars where neutral taste and shelf-life are paramount [97].

For high-water systems or lipid-rich spreads, (micro)emulsions provide high solubilization capacity and kinetic stability. For instance, a comparative work indicates that microemulsions can be more effective HXT carriers than conventional emulsions, while biocompatible water-in-oil microemulsions based on edible oils have been shown to support structured delivery and in vitro absorption advantages, useful for shots, oil-continuous dressings, or sauces [98].

These structural choices also interact with chemical stability because phenolics like HXT are susceptible to oxidation in aqueous and neutral pH environments (especially in the presence of dissolved oxygen and trace metals) [84]. In this sense, acidification, metal chelation, low-oxygen filling, and light/oxygen-barrier packaging are prudent defaults during the production of beverages, with ascorbate as a sacrificial antioxidant where label policy allows [84].

Furthermore, matrix effects on bioaccessibility should guide both product design and consumer directions. Static in vitro digestion models applied to olive-derived matrices report that protein- and fibre-rich contexts can reduce the bioaccessible fraction of HXT or tyrosol, which is consistent with polyphenol–protein and –fibre interactions [99,100]. By contrast, lipid phases and microemulsions tend to favour solubilization and protectants, while cyclodextrin complexes shift release toward intestinal conditions and simultaneously attenuate in-mouth bitterness [101]. In vitro digestion models and mechanistic studies show that protein- and fiber-rich matrices can decrease the bioaccessible fraction of HXT/tyrosol compared to lipidic or microstructured systems that promote solubilization and protection from early oxidation [101]. Reported effects include reduced release and micellar incorporation when phenolics interact with casein/whey proteins or soluble fibers, whereas oil-in-water microemulsions and cyclodextrin complexes tend to enhance bioaccessibility [101]. Consequently, claims and dosages should be studied by food matrix, and clinical protocols should standardize intake and postprandial sampling to minimize variability.

Putting these principles into concrete formats, several categories stand out. In baked goods, HT–α-cyclodextrin inclusion complexes have been dosed into wheat bread with acceptable sensory outcomes and measurable postprandial advantages, and the low water activity of the finished product is congenial to phenolic stability [86]. In lipid-based spreads and dressings, oil-continuous or microemulsion systems enable higher loading and oxidative buffering while maintaining palatability [98]. In acidified beverages and ready-to-drink products, combining encapsulation or inclusion with oxygen control and chelation sustains quality over shelf-life and keeps bitterness below recognition thresholds [85,101].

According to Web of Science, 242 documents were retrieved on 8 September 2025 using the search terms “hydroxytyrosol”, “application”, and “foods”. Forty-eight reviews were excluded and eleven items classified as proceedings papers, congresses abstract, or book chapters. In this regard, from these forty-eight reviews, eleven of them were partially focused on the application of olive by-products in industry, which demonstrates the real application of HXT extracts in food industry. Specifically, Silva et al. [96] reported 27 manuscripts referred to the applicability of HXT in edible oils, beverages, and vegetable-based, and bakery food products, all of them published between 2003 and 2019. Souilem et al. [102] reviewed eighteen publications, Gallardo-Fernández et al. [103] reported seven cases, Gullón et al. [104] six documents, Monteiro et al. [105] nine articles, Martínez-Zamora et al. [87] fourteen publications, Otero et al. [106] nine papers, and Difonzo et al. [107] fifty-seven scientific papers related to the use of olive leaf extract in food industry, which comprehensively covers all the practical applications of HXT in food products up to 2021.

Also, Madureira et al. [108] reviewed 16 scientific works and 21 patents related to the application of phenolics from olive pomace in industry. Particularly, several patents have been related to specific methods applied in the food industry and commercial reformulations based on phenolic compounds present in olives—CN117441778 about the storage method of dried chilies with HXT, US20220330558 about the use of HXT in bread, pasta, and baked goods in general, US 2019/012952 A1 about the method of producing beverages containing HXT without changing smell and flavor, and US 2010/0297330 A1 about the production of yogurts with HXT, among others.

Therefore, only publications from 2025 (*n* = 16), 2024 (*n* = 20), 2023 (*n* = 16), 2022 (*n* = 16), and 2021 (*n* = 14) were selected for the present review. Of these eighty-two scientific articles, only nine papers investigated the incorporation of HXT extract in food matrixes (Table 2), while the rest focused on the extraction, purification, and functionality of this compound. These data complete the last reviews recently published by Rodríguez-Pérez et al. [109] and Contreras-Angulo et al. [97] who have included three new publications from this period (2021–2025).

Table 2 outlines how HXT and related olive phenolics are being translated into real foods through four complementary routes: (i) transfer from EVOO by immersion [110], (ii) enzymatic modification of edible oils [111], (iii) valorization of olive by-products in beverages and baked goods [69,94,112,113,114], and (iv) incorporation into active packaging [115].

During oil-curing, extraction into foods is efficient (~80–90%), with HXT peaking faster in cheese and fish (~8 days) than in vegetables (~16 days), underscoring matrix-dependent kinetics [110]. One-step enzymatic esterification of oils using HXT-rich extracts achieves up to 81.2% recovery and markedly improves thermo-oxidative stability under accelerated conditions (60 °C, 28 days) [111].

In beverages, low-alcohol beer fortified with olive leaf extract shows higher total phenolics and polyphenols (~729 mg/L), together with enhanced antioxidant capacity [112]. Kombucha produced on olive by-product substrates accumulates substantial HXT (~30 mg/L) and oleuropein (~395 mg/L) [114]. A coffee–olive pomace functional brew increases phenolics and antioxidant activity (~6.62–8.17 mmol TE/L) [113]. Furthermore, the 10% of this formulation was most acceptable in a murine model and exhibited 30.5% α-amylase inhibition at 200 µg/mL, suggesting metabolic potential [113]. Beyond foods, chitosan or PVA films loaded with olive pomace extract (0.01–0.1% *w*/*v*) deliver antimicrobial activity (MIC 2.5 mg/mL against *E. coli*; ~10 mg/mL against *B. subtilis*), with HXT-tyrosol as dominant phenolics [115]. Spray-dried lecithin-based powders from olive leaf extracts provide quantifiable HXT (~42.60 mg/100 g) and improve cake quality at a 3% addition level [94].

Overall, the field is converging on ready-to-eat formats and functional packaging, while key gaps remain in dose standardization, process-resilience (bioaccessibility and bioavailability), sensory performance, and industrial stability testing (Figure 5). Therefore, in specification-setting and claims strategy, developers often use the EU regulatory anchor to calibrate internal targets, since according to the EFSA panel’s opinion that “olive-oil polyphenols contribute to the protection of blood lipids from oxidative stress” is allowed when the amount provided of HXT is at least 5 mg [18,20].

## 7. Future Research in Food Development and Clinical Perspectives

According to these scientific advances, future work should close the gap between promising mechanisms and claim-ready, scalable nutrition by treating delivery as part of the biology (Table 3). Since HXT is rapidly conjugated and its in vivo effects depend on the spatiotemporal profile of exposure, food development ought to progress hand-in-hand with pharmacokinetic–pharmacodynamic mapping. Focusing on this research field, the immediate priority is to compare two practical delivery families that are already manufacturable at scale: food matrices that leverage lipid phases and standardized supplement-style systems that favour rapid appearance and tunable persistence, such as free HXT, HXT-esters, nanoemulsions, micelles, cyclodextrin inclusion complexes, and polymeric microparticles. These comparisons should be executed under identical analytical control, report exact daily HXT dose (mg/day), capture the major Phase-II conjugates in plasma and urine, and disclose any co-actives (oleuropein, tyrosol, and phenolic esters) that may shape absorption or second-messenger signalling. Moreover, because matrix composition measurably alters HXT bioaccessibility (proteins, notably casein, and specific fibers can reduce the absorption of this compound during digestion, whereas lipid phases and microstructured carriers tend to protect and solubilize the molecule) trial protocols should specify the fed state, co-ingestion rules, and intake timing.

Food design research should prioritize three lines of work that directly enable credible clinical testing. First, optimize dose fidelity and sensory acceptability in finished products. Hydrolyzed leaf extracts or purified HXT should be the default inputs to avoid the harsh bitterness associated with oleuropein. When higher loads are needed, inclusion complexation and encapsulation can decouple in-mouth intensity from delivered dose. To enable robust dose–response modelling, future trials should pre-specify feeding state (fasted vs. postprandial), standardize co-ingestion (protein/fiber/fat), and report time-controlled dosing, along with the metabolite profile (free vs. conjugated forms). This will improve comparability across matrices and formulations and clarify the exposure–response relationship. Second, strengthen stability engineering for aqueous systems, where HXT is oxidation-prone under neutral pH, oxygen, and trace metals. Acidification, chelation, low-oxygen filling, and light/oxygen-barrier packaging should be standardized. Subsequently, perform shelf-life stress testing with concurrent potency and impurity monitoring, thereby ensuring that the administered dose equals the biologically delivered dose at consumption. Future studies should quantify 8-oxo-dG and strand-break endpoints alongside metal-chelation metrics, and deploy spectroscopic + in silico tools to test DNA surface binding vs. electrostatic surface association. Third, converge on shared analytics; an acid-hydrolysis-based assay for total HXT+tyrosol as the specification anchor, complemented by targeted LC-MS panels for principal conjugates (glucuronides and sulfates) and selected lipid peroxidation, nitration, and protein-oxidation markers. Harmonized methods will make cross-study synthesis possible and are essential if eventually seeking qualified claims beyond olive oil.

On the clinical side, a neuroprotective program for HXT now rests on convergent human data (vascular improvement, inflammatory down-tuning, and first clinical signals on cognition and psychomotor function) mapped to well-defined molecular pathways (Keap1–Nrf2–ARE, PI3K/Akt–ERK, AMPK–SIRT1–PGC-1α) to translate this into claim-ready nutrition science.

Currently, as described above, human trials indicate meaningful vascular/oxidative effects from ~7–15 mg HXT/day (chronic) to ~30–60 mg (acute) with good tolerability. High-phenolic EVOO typically delivers comparable HXT-equivalents when consumed at ~25–50 mL/day. Safety signals are favourable in adults and special populations studied so far, but trials should continue to monitor liver enzymes, ferritin/iron indices (given phenolic chelation potential), and interactors such as antithrombotics or MAO-active drugs.

For large-vessel endothelial function, include flow-mediated dilation, pulse-wave velocity, and the augmentation index. For BBB integrity, use dynamic contrast-enhanced magnetic resonance imaging and, where available, emerging BBB–ASL exchange metrics. Pair these measures with circulating vascular and BBB biomarkers, resting-state functional connectivity, and neurovascular-coupling readouts, together with a brief, validated battery assessing cognition and mood. Collect all endpoints at pre-specified time points, and track exposure in parallel via plasma and urine HXT conjugates (note alcohol as a DOPET confounder). Begin with cohorts featuring mild cognitive impairment, vascular cognitive-impairment risk, and mood symptoms. Plan 12–24 weeks for cognition/BBB outcomes and ≥6 months to detect progression signals and stratify analyses by baseline inflammatory load. Together, these steps outline a feasible path to link mechanistic signals with clinical trajectories in healthy and aging populations.

## 8. Conclusions

HXT has moved beyond theory to a credible neuroprotective candidate, with human data pointing to better vascular function, calmer inflammatory tone, and early gains in cognition and psychomotor performance. These effects align with well-known stress-response and energy pathways, which supports continued investment. What now matters most is not whether HXT works, but how precisely we deliver and measure it. Since HXT is quickly conjugated in the body, foods and supplements must be designed to control when and where active exposure occurs, and trials must verify that exposure with harmonized pharmacokinetic–pharmacodynamic readouts.

From a practical standpoint, current human data support chronic daily intakes of ~7–15 mg, while recognizing that the matrix/formulation and the reference risk modulate the magnitude of the effect. Explicit reporting of phenolic composition, co-ingestion, and timing will facilitate dose–response meta-inference and translation to neuroprotective applications. Furthermore, practical tools already exist to deliver reliable doses without sacrificing taste. Enzymatic or microbial hydrolysis of olive-leaf materials reduces bitterness at the source. Structuring strategies (cyclodextrin inclusion, polymeric microparticles, nano/microemulsions, and lipid-rich matrices) protect HXT from oxidation and separate mouthfeel from systemic delivery. Stability in aqueous systems can be engineered with acidification, chelators, low-oxygen filling, and barrier packaging. Matrix effects are real; proteins like casein and some fibers can decrease bioaccessibility, whereas lipid phases and microstructured carriers often improve it. Clear labelling and realistic use instructions should reflect these constraints.

Lastly, clinically, a claim-ready program should pair cognitive outcomes with a compact biomarker core of neurovascular and neuroimmune measures, sampled at baseline, early post-dose, and steady state. Doses around ~7–15 mg/day chronically and ~30–60 mg acutely are reasonable starting points, with high-phenolic EVOO offering a food-first route to similar exposures. Safety to date is reassuring, but continued monitoring of liver panels, iron indices, and plausible drug interactions is prudent. An integrated path can translate HXT from promising mechanism to validated, consumer-ready nutrition for brain health.

## Figures and Tables

**Table 1:** Summary of human studies of HXT-containing foods and supplements relevant to the neuroprotective effects of this compound.

Study Design	Population	Dose and Duration	Measured Parameters	Key Results	Ref.
Prospective, crossover, double-blind, placebo-controlled trial	Thirty adults with chronic coronary artery syndrome	HXT-enriched olive oil capsules (10 mg HXT/day); 1 month per period vs. control with crossover	Flow-mediated dilation, pulse wave velocity, perfused boundary region, coronary flow reserve, echocardiography, lipids, oxidized LDL, C-reactive protein	↑ Flow-mediated dilation, ↓ pulse wave velocity, ↓ perfused boundary region, ↑ coronary flow reserve; ↓oxidized LDL and C-reactive protein; integrated improvement in endothelial function and oxidative/inflammatory status	[65]
Randomized, single-blinded, dietary intervention	Sixty adults with overweight/obesity and type 2 diabetes mellitus	An amount of 60 g of whole wheat bread enriched with HXT vs. MD; 12 weeks	HbA1c, blood lipid levels, inflammatory markers, and weight loss	↓ body fat mass; ↓ fasting glucose, HbA1c and blood pressure; ↓ blood lipid, insulin, TNF-α and adiponectin	[66]
Randomized controlled clinical trial, part of LIPIMAGE cohort	Forty-eight older adults post-myocardial infarction and healthy controls; thirty-four completed	Total of 25 mL/day of olive oils differing in phenolic content (naturally HXT-rich vs. lower-phenolic); 26 weeks	Endothelial function, endothelial glycocalyx, oxidative stress indices; neuropsychiatric composite (reported)	Post-MI subgroup showed improved endothelial function and ↓ endothelial glycocalyx with high-phenolic EVOO; improved redox markers and signals on neuropsychiatric function	[67]
Crossover, randomized, double-blind and placebo-controlled clinical trial	Eighty-four adults with cardiovascular risk (completed sixty-seven)	Oral supplement with HXT and punicalagin; 20 weeks	Triglycerides, HDL-C, LDL-C, high-sensitivity C-reactive protein, metabolic syndrome z-score	↓ Triglycerides and LDL-C; ↑ HDL-C improved composite cardiometabolic risk (metabolic syndrome z-score)	[68]
Randomized, controlled, double-blind, and unicentric clinical trial.	Sixty-five adults with moderate hypercholesterolemia	Total of 100 g/day of BienStar^®^ cooked ham enriched with olive phenolics (including HXT) vs. placebo ham; 8 weeks	Endothelin-1, oxidized LDL, blood lipids and inflammatory markers	↓ systolic blood pressure and oxidized LDL; ↓ Malondialdehyde, ↓ total cholesterol, ↓ C-reactive protein, and ↓ interleukin 6	[69]
Randomised, controlled, parallel arm, clinical trial	Fifty adults undergoing with coronary angiography (completed 42)	Total of 25 mL/day EVOO vs. refined olive oil; 6 weeks	LDL-C, HDL-C, TG, CRP; cytokines; ex vivo LPS-stimulated	↓ LDL-C and CRP; reduced pro-inflammatory signalling and monocyte–endothelium adhesion with EVOO	[70]
Randomized, crossover, controlled trial	Thirty-three healthy adults (completed 32)	An amount of 135 mL white wine + 25 mg tyrosol capsules (endogenous bioconversion to HXT) vs. control	Endothelial function, HDL-C, antithrombin III, endothelin-1, homocysteine; PBMC inflammatory/oxidative gene expression	↑ HDL-C and antithrombin III; ↓endothelin-1 and homocysteine; down-regulated endothelial inflammation genes	[71]
Randomized, parallel, double-blind, placebo-controlled	Thirty-two adults with moderate hypercholesterolemia (LDL > 100 mg/dL and/or total cholesterol > 200 mg/dL)	Total of 7.5 mg HXT + 210 mg almond skin polyphenols per day; 8 weeks	Oxidized LDL; LDL-C, HDL-C, TG; IL-1β, IL-6, IL-10; safety	↓ Oxidized LDL Favourable cytokine modulation (↓ IL-1β/IL-6; ↑ IL-10)	[72]
Randomized, double-blind, placebo controlled crossover design	Fifteen healthy volunteers	An amount of 28 mL Olive fruit water phytocomplex (OliPhenolia^®^, HXT-rich) for 16 days (cohort)	Plasma HXT and conjugates (UPLC-MS/MS), antioxidant enzymes (SOD, CAT), MDA; exercise stress readouts	Good bioavailability; peak after 1 h consumption; ↑ SOD/CAT and ↓ MDA; mitigated exercise-induced oxidative stress	[73]
Randomized, controlled, and double-blinded trial	Twenty-two healthy adults	An amount of 25 g Phenolic-rich EVOO and common olive oil. Postprandial intervention (2 h after)	Oxidized LDL, malondialdehyde, triglycerides; leukocyte SOD1 and CAT expression	↓ Oxidized LDL/MDA/TG within hours; ↑ SOD1 and CAT expression; rapid antioxidant/vascular signal	[74]
Exploratory clinical trail	Twenty healthy adults	Olive pomace polyphenol complex 3.2 g as 8 capsules; single dose	Circulating phenolic metabolites; NO, PGE2, MMP13 in human chondrocytes	Post-intake serum ↓ NO, PGE2, MMP13; confirms resveratrol and HXT absorption and anti-inflammatory bioactivity	[75]
Randomized, parallel-group, double-blind, placebo-controlled trial	Forty adults at low–moderate cardiovascular risk (*n* = 20)	An amount of 12.5 g nutraceutical combination Aquilea Colesterol^®^ (olive-derived components including HXT) vs. placebo; 90 days	Inflammatory biomarkers, lipid profile, metabolic parameters	Improved inflammatory profile and cardiometabolic markers vs. placebo	[76]
Multicenter, randomized, observer-blinded trial	One hundred and nine patients >50 years diagnosed with unilateral exudative age-related macular degeneration (AREDS); ninety-three completed	AREDS-based supplement enriched with resveratrol and HXT (Retilut^®^ and Theavit^®^ for control group); 2 tablets/day; 12 months	Progression to neovascular age-related Macular Degeneration in the contralateral eye; visual function; inflammatory cytokines; fatty acid profile; and lutein-zeaxantin serum concentration.	Lower risk of contralateral neovascular Age related Macular Degeneration; improving fatty acid profile and increase carotenoid levels; improving proinflammatory and proangiogenic profile of patients	[77]
Randomized controlled clinical trial	Patients with mild Alzheimer’s disease; 55 enrolled, 23 completed	Olive leaf extract beverage (oleuropein/HXT-rich) vs. control MD; 6 months	MMSE, ADAS-Cog, CDR, functional scales, neuropsychiatric inventory, sleep	Maintained global cognition (MMSE) vs. decline in controls; improvements in neuropsychiatric symptoms thanks to olive leaf beverage consumption	[78]
Randomized, double-blind, placebo-controlled, parallel-group trial	Seventy-two participants (51–82 years old)	Total of 6 g of desert olive tree pearls per day (rich in HXT) vs. placebo; 12 weeks	Cognitrax test; cardiometabolic markers; safety	Improvements in memory composite and psychomotor speed vs. placebo; potential to alleviate cognitive problems; good adherence/safety	[79]
Randomized controlled trial (DIRECT-PLUS)	Two hundred and fifty-six adults (>30 years) with abdominal obesity or dyslipidemia	Dietary arms differing in polyphenol load (including high-polyphenol MD and physical activity); 18 months	DNA-methylation aging pace (DunedinPACE), urinary phenolics (HXT, tyrosol, and urolithin C), and cardiometabolic markers	↑ urinary HXT associated with slower epigenetic aging pace; supports systemic anti-aging signature	[80]
Randomized clinical trial, IMPACT BCN	Seventy-one high-risk pregnant women	MD enriched in EVOO and walnuts; 14 weeks pregnancy duration	Brain cortical thickness by magnetic resonance imaging; maternal diet adherence; urinary HXT	MD subgroup showed larger cortical-surface areas (precuneus, superior parietal); urinary HXT positively associated with maternal brain structure	[81]
Open-label, longitudinal pilot study	Nine participants 8–13 years with mitochondrial disease, especially with MELAS (Mitochondrial encephalopathy, lactic acidosis, and stroke-like episode)	Total of 10–30 mg HXT/day; 12 + 6 months	Pediatric Quality of Life and International pediatric mitochondrial disease scores, biochemical markers; tolerability; brain magnetic resonance imaging	Largest gains in quality of life; signals of benefit in MELAS subgroup; good tolerability	[82]

**Table 2:** Food applications of HXT and main findings from 2021 to 2025.

Food	Form and Dose	Analysis	Main Findings	Ref.
Fish (salmon and cod), vegetables (cherry tomatoes and eggplants), and cheese (curated and soft) oils in curated oil	Immersion in EVOO. Curation for 30 days in EVOO	Tyrosol, HXT, and oleuropein content during curation	The HXT extraction efficiency was about 80–90% in all tested foods. The concentration of HXT reached the maximum after 8 days in cheese and fish, while 16 days were needed to reach maximum values in vegetables	[110]
Pomace olive oil and sunflower oil	The extract was the substrate for the one-step enzymatic modification of selected oils	Antioxidant and enzymatic activity, fatty acid, volatile, and phenolic composition during accelerated oxidation (thermal treatment at 60 °C for 28 days)	The recovery of HXT reached up to 81.2%. The extract-modified oils presented notable thermos-oxidative stability at 60 °C for 28 days	[111]
Low-alcohol light beer	Olive leaf extract at 0.5, 1, and 2%	Physical-chemical quality, antioxidant activity and phenolics	The addition of the extract enriched the content of total phenolics (437.4 mg GAE/mL) and polyphenolic (729.0 mg/L)	[112]
Functional beverage from coffee and olive	Amounts of 5%, 10%, 15%, and 20% coffee olive pomace	Phenolics, antioxidant capacity. The 10% brew was used to measure the behaviour in a murine model	Adding coffee–olive powder to ground coffee increased the total phenol content in the brews. The highest antioxidant activity was 6.62–8.17 mmol TE/L. The 10% brew had the highest acceptance in mice, with increased consumption, greater exploratory behaviour, and reduced resting time. It also showed 30.5% α-amylase inhibition at 200 µg/mL	[113]
Kombucha	Olive mill by-products as a fermentation substrate (25, 50, and 100%)	Physical–chemical quality, antioxidant capacity, and phenolics	High content of HXT (29–9 mg/L) and oleuropein (395 mg/L)	[114]
Active packaging films	Chitosan or Polyvinyl alcohol with olive pomace extract (0.01–0.1%, *w*/*v*) films.	Antibacterial and antioxidant activity, phenolic content	Olive pomace extract had a minimum inhibitory activity of 2.5 mg/mL against *E. coli* and 10 mg/m against *B. subtilis.* HXT and tyrosol were identified as the major phenolic compounds	[115]
Cake formulations	Lecithin-olive leaf extracts spray-dried using maltodextrin and whey protein at 1–3%	Physical–chemical quality, antioxidant capacity, and phenolics	The HXT and tyrosol contents of the powder were 42.60 ± 4.51 mg/100 g and 15.48 ± 2.50 mg/100 g, respectively. The addition of olive leaf extracts at a concentration of 3% to the powder cake premix positively affected the final product properties	[94]
BienStar^®^ cooked ham	A total of 4.45 mg HXT/100 g cooked hamb	Blood lipids and inflammatory markers in 65 adults with moderate hypercholesterolemia in a randomized, controlled, double-blind clinical trial	↓ systolic blood pressure and oxidized LDL; ↓ Malondialdehyde, ↓ total cholesterol, ↓ C-reactive protein, and ↓ interleukin 6	[69]

**Table 3:** Translational framework (from molecule to clinic).

Component	Translational Implication
Physicochemistry → Exposure	Limited passive permeability + rapid conjugation → guides dose and frequency
Matrix/formulation	Lipid carriers/microstructures ↑ bioaccessibility
	Protein/fiber-rich matrices ↓ bioaccessibility
Target engagement	Pair systemic conjugates with endothelial redox and neuroinflammation biomarkers
Population	Enrich higher oxidative/inflammatory baseline cohorts
Trial design	Pre-specify fed/fasted
	Co-ingestion
	Sampling windows
	Consider efflux/transporters
Outcomes	Combine vascular/neuroimmune endpoints with cognitive/neuromotor measures

## Data Availability

No new data were created or analyzed in this study. Data sharing is not applicable to this article.

## Abbreviations

6-OHDA: 6-hydroxydopamine; ADAS-Cog: Alzheimer’s Disease Assessment Scale—Cognitive Subscale; Akt/PKB: Protein kinase B; ALT: Alanine aminotransferase; AMPK: AMP-activated protein kinase; Apo A1: Apolipoprotein A1; Apo B: Apolipoprotein B; ARE: Antioxidant Response Element; AST: Aspartate aminotransferase; BDNF: Brain-Derived Neurotrophic Factor; CDR: Clinical Dementia Rating; CFR: Coronary flow reserve; CK: Creatine kinase; COX-2: Cyclooxygenase-2; CREB: Cyclic AMP response element-binding protein; DCE-MRI: Dynamic Contrast-Enhanced Magnetic Resonance Imaging; eNOS: Endothelial nitric oxide synthase; ERK: Extracellular signal-regulated kinase; FMD: Flow-mediated dilation; FRSSD: Functional Rating Scale for Dementia; FUCAS: Functional Cognitive Assessment Scale; GDS: Geriatric Depression Scale; GGT: Gamma-glutamyl transpeptidase; GSDMD: Gasdermin D; HDL: High-density lipoprotein; hCMEC/D3: Human cerebral microvascular endothelial cells, clone D3; HRQoL: Health-Related Quality of Life; ICAM-1: Intercellular Adhesion Molecule-1; IL-1β: Interleukin-1 beta; IL-6: Interleukin-6; JAK: Janus kinase; LDH: Lactate dehydrogenase; LDL: Low-density lipoprotein; LPS: Lipopolysaccharide; MMP-13: Matrix metalloproteinase-13; MMSE: Mini-Mental State Examination; MyD88: Myeloid differentiation primary response 88; NF-κB: Nuclear factor kappa-light-chain-enhancer of activated B cells; NF-κB/MAPK signalling: Nuclear factor κB/Mitogen-activated protein kinase signalling; NLRP3: NOD-like receptor family, pyrin domain-containing 3; NO: Nitric oxide; NOX2: NADPH oxidase 2; NPI: Neuropsychiatric Inventory; NQO1: NAD(P)H quinone dehydrogenase 1; Nrf2: Nuclear factor erythroid 2-related factor 2; NOS2: Inducible nitric oxide synthase; PGC-1α: Peroxisome proliferator-activated receptor gamma coactivator 1-alpha; PGE2: Prostaglandin E2; PI3K: Phosphoinositide 3-kinase; PWV: Pulse wave velocity; SIRT1: Sirtuin 1; SODs: Superoxide dismutases; STAT: Signal Transducer and Activator of Transcription; TLR: Toll-like receptor; TNF-α: Tumor necrosis factor-alpha; VCAM-1: Vascular Cell Adhesion Molecule-1; α-syn: Alpha-synuclein.
